# The Association Between Child and Family Characteristics and the Mental Health and Wellbeing of Caregivers of Children with Autism in Mid-Childhood

**DOI:** 10.1007/s10803-017-3392-x

**Published:** 2017-11-25

**Authors:** Erica Salomone, Kathy Leadbitter, Catherine Aldred, Barbara Barrett, Sarah Byford, Tony Charman, Patricia Howlin, Jonathan Green, Ann Le Couteur, Helen McConachie, Jeremy R. Parr, Andrew Pickles, Vicky Slonims, The PACT Consortium, Rachel Cole-Fletcher, Isobel Gammer, Jessica Maxwell, Hannah Tobin, George Vamvakas

**Affiliations:** 1 University of Turin, Via Po, 14, 10123 Turin, Italy; 2 Institute of Psychiatry, Psychology & Neuroscience, King’s College London, London, UK; 3 University of Manchester, Manchester, UK; 4 UK Royal Manchester Children’s Hospital, Manchester Academic Health Sciences Centre, Manchester, UK; 5 Greater Manchester Mental Health NHS Foundation Trust, Manchester, UK; 6 Newcastle University, Newcastle, UK; 7 Northumberland, Tyne and Wear NHS Foundation Trust, Newcastle upon Tyne, UK; 8 Evelina London Children’s Hospital, Guys and St Thomas University NHS Trust, London, UK

**Keywords:** Autism, Mental health, Mental wellbeing, Caregiver, Daily living skills, Emotional and behavioural difficulties

## Abstract

We examined predictors of mental health difficulties and wellbeing in caregivers of children with autism in the Pre-school Autism Communication Trial cohort in middle childhood (N = 104). Child’s intellectual disability, daily living skills impairment, elevated emotional and behavioural difficulties, high educational level of caregiver and household income below the median significantly predicted caregivers’ mental health difficulties, but autism severity, child communication skills and family circumstances did not. Lower caregiver mental wellbeing was predicted by elevated child emotional and behavioural difficulties. The need to support the mental health and wellbeing of caregivers of children with autism is discussed in light of the results.

## Introduction

Among parents of children with autism, higher rates of mental health problems and stress are reported compared to parents of children with other developmental or physical disabilities (Olsson and Hwang 2001; Cohrs and Leslie 2017; Hayes and Watson 2013; Abbeduto et al. 2004). Such increased rates have been termed as the ‘ASD disadvantage’, to indicate that poorer mental health in the caregiver may be directly related to the child’s diagnostic features (Hartley et al. 2012). There are indeed several characteristics of children with autism that can exacerbate parenting difficulties, increase stress and reduce wellbeing. These include ‘core’ autism symptoms (e.g. social communication impairments, interfering restrictive/repetitive behaviours), but also poor cognitive and adaptive skills, and the emotional and behavioural difficulties that are frequently co-occurring features. However, research investigating the association between child autism severity and the mental health of caregivers has yielded mixed results. Thus, although some studies (Benson 2006; Hastings and Johnson 2001; Schieve et al. 2011) report a positive association with stress, others do not (McStay et al. 2014; Hastings et al. 2005). Similarly, while impairments in adaptive behaviour, and specifically daily living skills (DLS), are key areas of deficit in autism, some studies have reported a relationship between better DLS and lower parenting stress (Tomanik et al. 2004; Green and Carter 2014) and others have found no association (Lecavalier et al. 2006; Estes et al. 2009, 2013; Peters-Scheffer et al. 2012). For overall adaptive behaviour, both a negative association (Hall and Graff 2011) and lack of association (Feldman et al. 2007) with parental stress have been described. Cognitive impairment frequently co-occurs in autism (CDC 2014), but the association of child cognitive functioning with parental outcomes is not fully understood. Poorer parental outcomes have been reported to be associated with both intellectual disability (ID) (Baker-Ericzén et al. 2005) and higher intellectual functioning (Rivard et al. 2014), while other studies did not report any association (Peters-Scheffer et al. 2012; Estes et al. 2013). In contrast, data on the association between higher rates of child emotional and behavioural problems and poorer parental psychological well-being are more consistent (Estes et al. 2013; Lecavalier et al. 2006; Peters-Scheffer et al. 2012; Hartley et al. 2012). Among family factors, social support including support from a partner (Ekas et al. 2010; Dunn et al. 2001; Bromley et al. 2004) may act as buffers or protective factors. In contrast, family disadvantage, such as parenting multiple children with disabilities (Orsmond et al. 2007) and being a single parent (Olsson and Hwang 2001) may exacerbate stress and mental health difficulties. With regard to socioeconomic factors, lower parental education and income have been found, in some cases, to be associated with higher rates of common mental health difficulties and poorer psychological wellbeing in a general population sample (Jones and Nicolás 2004). Finally, child’s age may be a moderating factor in the relationship between child and family characteristics and parental psychological outcomes. Thus, there is some evidence that the maturational changes that occur in middle childhood combined with children’s increased exposure to social situations require major adjustments in parental expectations which, in turn, may be associated with higher risk for stress compared to both early years and adolescence (Orr et al. 1993).

In summary, while there is substantial evidence of a relationship between child and family characteristics and parental psychological outcomes, the interpretation of apparent discrepancies in the findings is made difficult by the fact that such factors are often examined in isolation, and not within more comprehensive models. Additionally, the outcomes considered include a range of constructs, from mental health difficulties, to stress and reduced mental wellbeing. This methodological issue reflects a broader conceptual problem in measuring psychological outcomes, that is, the question of the degree of overlap between the absence of mental health symptoms and good psychological well-being. These constructs are often correlated but have evidence of distinct antecedents (Kinderman et al. 2015).

In light of these considerations, this study focuses on mental health difficulties *and* mental wellbeing in caregivers of children with autism in mid-childhood, examining the association of both with a range of child and family factors. The study is a secondary analysis of data from the follow-up to the Pre-school Autism Communication Trial (PACT) (Pickles et al. 2016). PACT was a two-arm single-blind randomised controlled trial comparing a parent-mediated, communication focussed intervention with treatment as usual; at the original recruitment, children were between 2 and 4 years 11 months and met criteria for ‘core autism’ (Green et al. 2010). Here we examine rates of mental health difficulties and mental wellbeing in the parents at the 6-year follow-up. We explore the relationship with child factors (autism severity, language, intellectual disability (ID), daily living skills, emotional and behavioural difficulties) and family factors (income, level of education, being a single parent, parenting multiple children with developmental difficulties, PACT therapy in pre-school years). Based on the available evidence, we hypothesise that mental health and mental wellbeing would be lower in caregivers of children with elevated emotional and behavioural difficulties. In light of mixed findings for the other factors, we do not pre-specify further hypotheses.

## Methods

### Participants

Participants were caregivers enrolled in the PACT randomised controlled trial (Green et al. 2010). The 12-month intervention focused on modifying the parent–child interaction in order to enhance child communication (Aldred et al. 2010, PACT Intervention procedure). The PACT trial and follow-up study (Pickles et al. 2016) were approved by the Central Manchester Multicentre Research Ethics Committee (Manchester, UK); further information on the studies is available online (http://research.bmh.manchester.ac.uk/pact/about). The current analysis is based on those caregivers who completed measures of mental health and mental wellbeing (see “Measures”) at the follow-up ascertainment when their children were aged 8–12 years. None of these parents had been identified as having severe psychiatric illness when the child was aged 2–4 years since this was an exclusion criterion for the trial. The total number of caregivers was 104 (86% of those seen at the follow-up; 68% of the original cohort); of these 49 (47.1%) had been assigned to the treatment arm in the original trial. Median caregiver age was 41 years; almost all respondents were mothers (n = 96, 92.3%); 60% (n = 61) were White British; 17% (n = 17) Black African; 8% (n = 8) Asian; 15% (n = 15) of other ethnic background.

### Measures

#### Mental Health

The General Health Questionnaire-12 items (GHQ-12, Goldberg 1992) was used to measure mental health difficulties. The GHQ-12 is sensitive to short-term psychiatric conditions and focuses on the inability to carry out normal functions (e.g. ‘not been able to concentrate on what you’re doing’) and psychological distress (‘feeling unhappy or depressed’). The ‘GHQ-scoring’ method, or ‘caseness’ approach to scoring was used. Items are rated as 1 if the response is ‘less than usual’ or ‘much less than usual’ (and rated as 0 otherwise), producing a possible Total score ranging from 0 to 12. As recommended in the WHO study (Goldberg et al. 1997), the screening cut-off was set at ≥ 2 on the Total score to define presence of mental health difficulties, or ‘caseness for mental health difficulties’ (hereafter, ‘GHQ-12 caseness’, as is general in the literature). As per guidelines, missing items were recoded as zero scores (there was 1 missing item for 1 participant).

#### Mental Wellbeing

The Warwick-Edinburgh Mental Wellbeing Scale questionnaire (WEMWBS, Tennant et al. 2007) was used to measure mental wellbeing. The 14 items, rated from 1 (‘none of the time’) to 5 (‘all of the time’), are positively worded, represent positive attributes of wellbeing and cover both feeling (e.g., ‘I’ve been feeling confident’) and functioning (e.g., ‘I’ve been dealing with problems well’). Following the authors’ recommendation, the Total wellbeing score, calculated as the sum of the ratings on each of the 14 items, was prorated for 7 participants (each of whom had 1 missing item).

#### Child Factors

Autism severity was assessed with the Autism Diagnostic Observation Schedule Second Edition (ADOS-2, Lord et al. 2012). The ADOS-2 Calibrated Severity Scores (CSS) for Social Affect (SA) and Restricted and Repetitive Behaviours (RRB) were computed and provide standardised ASD severity for the two domains based on the Module administered and the participant’s age and verbal ability (Hus et al. 2014). Participants were classified by level of speech as per the ADOS-2 Module administered, producing four categories of level of speech: Module 1 no words/Module 1 some words/Module 2 phrase speech/Module 3 fluent speech. Cognitive functioning and adaptive behaviour were respectively assessed with the British Ability Scales Third Edition (BAS-3, Elliott and Smith 2011) and the Vineland Adaptive Behavior Scales II Daily Living Skills (DLS) domain (VABS II, Sparrow 2005). Emotional and behavioural difficulties were measured by parent report using the Strengths and Difficulties Questionnaire (SDQ; Goodman 1997), a 25-item scale comprised of hyperactivity, emotional symptoms, conduct problems and peer problems subscales (as well as pro-social). Scale scores can be prorated if at least three items in each subscale are completed and UK normed screening cut-offs are available to identify ‘abnormal’ (top 10%) and ‘borderline’ (the next 10%) levels of difficulties by age and gender (http://www.sdqinfo.com). Given the expected elevated rates of emotional and behavioural difficulties on the SDQ in school-age children with ASD (Salomone et al. 2014), the ‘borderline’ and ‘typical’ categories were collapsed for the purposes of this analysis.

#### Family Factors

Household income was collected for both carers, where applicable, if they were sharing income (even if they were not living together). Participants indicated the category most appropriate for their income (ranging from < £5000 to > £100,000). A ‘household income’ value was calculated by taking the middle point of each income category and summing across both carers where applicable. The median household disposable income reported by the UK National Office for Statistics (ONS) for the financial year ending 2015 (£25,700; ONS 2016) was used to create a dummy variable for household income (below/equal and above the median). Caregivers indicated their educational level on the 8-level UK National Qualifications Framework (NQF). The number of people living in the house and the presence of developmental difficulties or disorder in siblings of the target child were recorded; caregivers self-identified ethnicity.

### Data Analysis

To investigate the association of child and parental characteristics with caregiver mental health difficulties and mental wellbeing, we conducted a multiple logistic regression and a multiple linear regression with, respectively, GHQ-12 ‘caseness’ (i.e., above GHQ-12 cut off for mental health difficulties) and WEMWBS Total score as dependent variables. Odds ratios (OR) and 95% confidence intervals (CIs) are presented for the logistic regression, and the unstandardized and standardized coefficients for the multiple linear regression.

Variables for child’s functioning (cognitive level [BAS Standard Nonverbal Composite (SNC)]; daily living impairment [VABS DLS Standard Score (SS)]; emotional and behavioural difficulties [SDQ Total problems]) and socioeconomic factors (income and educational level) were dichotomised into clinically meaningful subgroups in order to better capture their significance, as well as to facilitate the interpretation of the parameters (Ragland 1992).

The predictors in each model were child’s age (years), social affect (ADOS-2 SA CSS), restrictive and repetitive behaviours (ADOS-2 RRB CSS), level of speech (Module 1 no words/Module 1 some words/Module 2 phrase speech/Module 3 fluent speech), presence of intellectual disability (ID; BAS SNC ≤ 70), presence of DLS impairment (VABS DLS ≤ 70), presence of elevated emotional and behavioural difficulties (SDQ Total problems score ‘abnormal’), parental age (years), low educational level (NQF ≤ 4), low household income (below median), being a single parent, presence of other children with developmental disorders, PACT therapy in pre-school years.

## Results

### Child and Family Characteristics

Table 1 presents a summary of child and caregiver characteristics and Table 2 provides rates of GHQ-12 ‘caseness’ and WEMWBS scores by each factor.

**Table 1:** Participants’ characteristics

	N	n (%)	M (SD)	Range
*Child characteristics*				
Age (years)	104		10.55 (0.79)	8.17–12.67
Males	104	94 (90.4%)		
Autism severity (ADOS-2)	99			
Total (CSS)			6.62 (1.84)	1–9
Social affect (SA CSS)			7.18 (1.98)	1–10
Restrictive and repetitive behaviours (RRB CSS)			8.26 (1.69)	1–10
Level of speech (ADOS-2 module administered)	99			
Module 1 few/no words		25 (25.3%)		
Module 1 some words		12 (12.1%)		
Module 2 phrase speech		17 (17.2%)		
Module 3 fluent speech		45 (45.5%)		
Cognitive ability (BAS SNC SS)	101		66.05 (22.86)	40–124
Adaptive behaviour	98			
Socialization skills (VABS SOC SS)			58.63 (13.82)	42–112
Communication skills (VABS COMM SS)			63.86 (15.86)	38–120
Daily living skills (VABS DLS SS)			64.33 (13.83)	40–112
Emotional and behavioural difficulties (SDQ Total)	89		18.53 (5.42)	5–35
*Family characteristics*				
Age (years)	88		41 (6.39)	28–60
Ethnicity	101			
White British		61 (60.4%)		
Black African		17 (16.8%)		
Asian		8 (7.9%)		
Other ethnic background		15 (14.8%)		
Low educational level (NQF ≤ 4)	94	54 (57.4%)		
Household income below median (< £25,700)	102	57 (55.9%)		
Single parent	97	23 (23.7%)		
Other children with dev. diff./dis. in the house	97	30 (30.9%)		

*ADOS-2 CSS, SA CSS and RRB CSS* Autism Diagnostic Observation Schedule Second Edition Composite Severity Score for Total, Social Affect and Restrictive and Repetitive Behaviours, *BAS SNC SS* British Ability Scales Standard Nonverbal Composite Standard Score, *VABS SOC SS, VABS COMM SS and VABS DLS SS* Vineland Adaptive Behavior Scales Second Edition Standard Score for the Socialization, Communication and Daily Living Skills Domains, *SDQ Total* Strengths and Difficulties Questionnaire Total Problems Score, *NQF* National Qualifications Framework, *dev. diff/dis*. developmental difficulties/disorder

**Table 2:** Rates of mental health difficulties (GHQ-12 ‘caseness’) and wellbeing levels (WEMWBS Total) by child and family characteristics

	N = 104	N	GHQ-12 caseness		WEMWBS total	
			n	%	M	SD
*Child characteristics*						
Social affect						
ADOS-2 SA CSS high		49	23	46.9	46.73	10.30
ADOS-2 SA CSS minimal/low/moderate		50	25	50	46.73	8.19
Restrictive and repetitive behaviours						
ADOS-2 RRB CSS high		70	35	50	46.80	9.24
ADOS-2 RRB CSS minimal/low/moderate		29	13	44.8	46.57	9.41
Level of speech						
Module 1 few/no words		25	11	44	47.17	9.85
Module 1 some words		12	6	50	42.68	9.29
Module 2 phrase speech		17	10	58.8	43.72	10.89
Module 3 fluent speech		45	21	46.7	48.70	7.79
Cognitive ability						
BAS ≤ 70		65	36	55.4	45.95	9.87
BAS ≥ 71		36	14	38.9	47.88	7.76
Daily living skills						
VABS DLS ≤ 70		70	38	54.3	45.24	9.42
VABS DLS ≥ 71		28	9	32.1	49.80	7.67
Emotional and behavioural difficulties						
SDQ total abnormal		52	31	59.6	44.53	9.03
SDQ total typical/borderline		37	12	32.4	49.42	7.61
*Family characteristics*						
Household income						
Below median		57	29	50.9	45.07	9.79
Above median		45	19	42.2	48.91	7.97
Educational level						
Low (NQF ≤ 4)		54	24	44.4	47.03	9.51
Intermediate/high (NQF ≥ 5)		40	20	50	45.99	8.42
No. of parents living in the house						
1 parent		23	12	52.2	44.14	8.49
2 parents		74	34	45.9	47.20	9.21
Other children with dev. diff./dis.						
≥ 1 child		30	12	40	47.00	9.66
None		67	34	50.7	46.24	8.89
PACT therapy in pre-school years						
No		55	30	54.5	46.56	9.51
Yes		49	20	40.8	46.81	8.77

*ADOS-2 CSS, SA CSS and RRB CSS* Autism Diagnostic Observation Schedule Second Edition Composite Severity Score for Total, Social Affect and Restrictive and Repetitive Behaviours, *BAS SNC SS* British Ability Scales Standard Nonverbal Composite Standard Score, *VABS SOC SS, VABS COMM SS and VABS DLS SS* Vineland Adaptive Behavior Scales Second Edition Standard Score for the Socialization, Communication and Daily Living Skills Domains, *SDQ Total* Strengths and Difficulties Questionnaire Total Problems Score, *NQF* National Qualifications Framework, *dev. diff/dis*. developmental difficulties/disorder, *PACT* Pre-school Autism Communication Trial

### Mental Health Difficulties and Mental Wellbeing

A total of 50 parents (48%) met criteria for GHQ-12 ‘caseness’. A Wilcoxon signed-rank test indicated that the median mental wellbeing level of the sample (Mdn = 45, min: 25, max: 66) was lower than the median of the general UK population for the 35–44 age range (Mdn = 51, Z = − 4.344, *p* < .001). There was a strong negative correlation between the GHQ-12 Total score and the WEMBS Total score (Spearman’s rho = − 0.65, *p* < .001) as in the general population validation study (Spearman’s rho = − 0.53; Tennant et al. 2007). Caregivers who met GHQ-12 criteria for ‘caseness’ (M = 41.81, SD = 7.95) reported significantly lower mental wellbeing levels than caregivers who fell below the GHQ-12 cut-off (M = 51.18, SD = 7.77; t (92) = − 6.08, *p* < .001, Cohen’s d = 1.19).

Table 3 presents the results of the multiple logistic and multiple linear regressions. The logistic regression model’s χ^2^ and Hosmer and Lemeshow goodness-of-fit test statistics were respectively significant (χ^2^ = 31.187, *p* = .008) and not significant (χ^2^ = 4.384, *p* = .821), both indicating a well-fitting model; the Nagelkerke’s *R* ^2^ was moderate (0.459). Among child characteristics, ID, DLS impairment and abnormal-range SDQ Total score significantly predicted parent mental health difficulties [respectively: OR = 4.71, *p* = .047, 95% CI = (1.02, 21.77); OR = 12.03, *p* = .015; 95% CI = (1.61, 90.07) and OR = 4.20, *p* = .041; 95% CI = (1.06, 16.68)] over and above the effect of the other variables. Taking into account the effect of all other variables, parents who reported a household income below the population median were more likely to meet ‘caseness’ on the GHQ-12, as were parents with a relatively higher education level [respectively: OR = 16.16, *p* = .005, 95% CI = (1.57, 166.20); and OR = 0.07, *p* = .005; 95% CI = (0.01, 0.45)]. There was no association between GHQ-12 ‘caseness’ and the remaining variables. WEMWBS Total was predicted by elevated child emotional and behavioural difficulties (β = − 0.31, *p* = .011) alone. The overall model fit was *R* ^2^ = 0.246 (Table 3).

**Table 3:** Predictors of caregiver mental health difficulties and mental wellbeing

	GHQ-12 caseness		WEMWBS total score			
	OR [95% CI]	p*	B	SE B	β	p*
*Child*						
Age (years)	0.72 [0.33–1.60]	.427	0.73	1.32	0.07	.586
Social affect (ADOS-2 SA CSS)	0.80 [0.56–1.15]	.231	− 0.08	0.57	− 0.02	.892
Restrictive and repetitive behaviour (ADOS-2 RRB CSS)	0.72 [0.48–1.07]	.107	0.57	0.63	0.11	.367
Level of speech (reference category: Module 3 fluent speech)	–	–	–	–	–	
Module 1 few/no words	0.24 [0.02–2.22]	.208	− 0.73	3.49	− 0.04	.834
Module 1 some words	0.28 [0.02–4.24]	.358	− 2.79	4.47	− 0.09	.535
Module 2 phrase speech	0.96 [0.14–6.57]	.970	0.02	3.30	0.00	0.995
Intellectual disability (BAS SNC < 70)	**4.71** [**1.02–21.77**]	.**047**	− 0.67	2.46	− 0.04	.787
Daily living skills impairment (VABS DLS SS < 70)	**12.03** [**1.61–90.07**]	.**015**	− 4.01	2.85	− 0.22	.164
Emotional and behavioural difficulties (SDQ total: abnormal range)	**4.20** [**1.06–16.68**]	.**041**	− **5.43**	**2.09**	− **0.32**	.**012**
*Caregiver*						
Age (years)	1.02 [0.91–1.13]	.741	− 0.08	0.19	− 0.05	.678
Educational level (low: NQF < 5)	**0.07** [**0.01–0.45**]	.**005**	1.73	2.46	0.10	.486
Income (below median)	**16.16** [**1.57–166.20**]	.**019**	− 3.70	3.02	− 0.22	.226
Parents living in the house (one parent)	3.21 [0.51–20.00]	.212	1.72	3.03	0.09	.573
Other children with developmental disorders (1 or more)	0.49 [0.13–1.89]	.301	1.23	2.24	0.07	.585
Group (PACT therapy)	0.48 [0.12–1.99]	.312	− 0.42	2.22	− 0.02	.851

*ADOS-2 CSS, SA CSS and RRB CSS* Autism Diagnostic Observation Schedule Second Edition Composite Severity Score for Total, Social Affect and Restrictive and Repetitive Behaviours, *BAS SNC SS* British Ability Scales Standard Nonverbal Composite Standard Score, *VABS SOC SS, VABS COMM SS and VABS DLS SS* Vineland Adaptive Behavior Scales Second Edition Standard Score for the Socialization, Communication and Daily Living Skills Domains, *SDQ Total* Strengths and Difficulties Questionnaire Total Problems Score, *NQF* National Qualifications Framework

*Predictors significant at *p* < .05 are marked in bold

## Discussion

We examined predictors of mental health difficulties and mental wellbeing in a sample of caregivers of children with autism aged 8–12 years. Measuring mental wellbeing alongside mental health difficulties is increasingly considered important for outcome evaluation from users’ perspectives; however, it is unclear whether they truly represent two distinct *continua*. In the general population, there is evidence that mental health and mental wellbeing have different determinants, with mental health problems for instance associated with adverse life events and wellbeing more associated with social circumstances (Kinderman et al. 2015).

Confirming previous reports, the results demonstrate that raising a child with autism is associated with an increased risk for both mental health difficulties and reduced wellbeing. Thus, in our sample 48% of caregivers fell above the GHQ-12 cut-off for mental health difficulties, while 24% is the expected rate in the general population (Goldberg et al. 1997); median wellbeing levels on the WEMWBS were also significantly lower than the expected UK population median (Tennant et al. 2007; Maheswaran et al. 2012). However, when we examined the overall estimated probability of meeting ‘caseness’ on the GHQ-12, neither child core autism symptoms (social affect and restrictive and repetitive behaviours), nor child level of speech, were associated with caregiver mental health difficulties. We found instead that prevalence of GHQ-12 ‘caseness’ was significantly higher in caregivers of children with ID and/or poor daily living skills and in those with elevated child emotional and behavioural difficulties. Child behavioural difficulties in autism are, across all age ranges, from toddlerhood to young adulthood, associated with parent mental health difficulties (Estes et al. 2013; Lecavalier et al. 2006; Peters-Scheffer et al. 2012) and our results replicate these findings. We also found these to be the only predictive factor for caregiver wellbeing. Low levels of wellbeing are associated with social isolation in the general population (Kinderman et al. 2015), and it possible that child behaviour difficulties lead to greater isolation and impact on caregivers’ overall functioning, over and above other child and family factors. Moreover, child emotional and behavioural difficulties was the only factor associated with both mental health difficulties and reduced wellbeing, which is notable because the GHQ-12 and the WEMWBS were strongly correlated. While the concurrence may be partly attributable to rater effects as the same caregiver completed both questionnaires at the same point in time, the different patterns of association with the potential risk factors may reflect the fact that in at-risk populations the two constructs may *de facto* overlap more than in the general population, representing commonly co-occurring outcomes with different determinants. This point is exemplified by the WHO definition where mental health is equated to a state of well-being when the individual cope[s] with the *normal* stresses of life (Italics added; Herrman et al. 2005, p. XVIII).

The pattern of findings also indicates that neither parental mental health nor psychological wellbeing are *directly* affected by the severity of autism per se. Thus, while some studies comparing caregivers of children with ASD or other developmental disorders have suggested a disorder-specific effect, others find that group differences in parental outcomes are confounded by differences in child comorbidities or characteristics, rather than being related to core diagnostic criteria (Griffith et al. 2010; Hartley et al. 2012). Similar effects have been shown for caregivers of children with developmental delay, where higher stress was attributable to the extent of behaviour problems rather than to the presence of the developmental delay itself (Baker et al. 2003). The evidence on the effect of cognitive impairment on parental outcomes in caregivers of children with autism is inconclusive. In our sample, where the majority of children had IQ ≤ 70 (while the estimated rate of co-occurrence is 31%, CDC 2014), ID was significantly associated with GHQ-12 caseness. Our finding of poor child daily living skills predicting mental health difficulties, over and above the effect of ID, adds to the existing mixed evidence (Estes et al. 2013; Green and Carter 2014) and may be due to the age of the children in our sample. As previously suggested (Estes et al. 2013), it may be easier for caregivers to compensate for poor daily living skills when children are younger, because they are physically more manageable and the gap with peers is not as wide as in mid-childhood (Fisch et al. 2002; Gabriels et al. 2007), particularly in children with co-occurring ID (Hill et al. 2015).

Although partner support (Ekas et al. 2010) and having several children with disabilities in the family (Orsmond et al. 2007) have been found to be correlates of psychological outcomes in other studies, we did not find presence of another carer or number of other children with developmental difficulties to be predictive. Above and beyond the effect of child characteristics, the odds of caregivers reporting mental health difficulties were significantly increased by low household income, but decreased by low educational level, although the magnitude of the effect was more limited for the latter. Our results align with previous reports of adversity as a risk factor; however it should be noted that the association between socio-economic status and GHQ-12 ‘caseness’ is inconsistent (Laaksonen et al. 2007). In the WHO study, the GHQ-12 was not influenced by educational level *per se* (Goldberg et al. 1997), but in our study, over and above the effect of other variables, parents with low educational level were slightly less likely to meet ‘caseness’ for mental health difficulties. This may reflect multiple factors, such as under-reporting of symptoms in caregivers with low educational level due to difficulties in verbal comprehension, as highlighted by Kessler and Üstün (2004) or other contextual factors associated with work commitments in highly educated caregivers (Parkes et al. 2015). Finally, it is worth mentioning that pre-school assignment to PACT intervention was not associated with rates of parental mental health difficulties or mental wellbeing levels when the children were in middle childhood. The PACT trial, which did not include parental psychological outcomes, has been shown to have lasting treatment effects on increased child initiations and in reduced severity of autism symptoms (Pickles et al. 2016) as well as on positive family life experience, as measured by the Autism Family Experience Questionnaire (AFEQ) (Leadbitter et al. in press). However in this study we did not find a protective effect of PACT over and above the effect of all the other risk factors, suggesting that the benefits have not carried over into more general amelioration of stresses upon caregivers of autistic children six years later. Other autism intervention trials, such as Kasari et al. (2015) and Estes et al. (2014), have reported positive effects on parenting stress, but with a much shorter follow-up periods and with a specific focus on parenting stress, rather than general mental health and wellbeing. Further research could inform whether such short-term intervention-related reductions in parental stress are sustained over the longer term and linked to overall mental health and wellbeing.

The study was limited by the lack of baseline measurement for mental health and mental wellbeing, which prevented the analysis of longitudinal associations. Additionally, there are several potential risk factors (such as negative life events, family conflict, medical illness) and protective factors (such as social support, adaptive coping strategies, self-efficacy) for poor mental health and mental wellbeing (WHO 2012) that were not measured and might have added to the predictive strength of the study. The moderate sample size required us to exclude from the analysis further subsidiary predictor variables, such as ethnicity, that may be associated with mental health status. Finally, for ease of interpretation, we opted for the ADOS Module classification as a measure of functional language in a sample where the majority of children had basal scores on formal language tests; this precluded a more fine-grained analysis of language impairment as a predictor variable. Strengths include the rigorous characterization of the sample and the combination of measures for parental psychological outcomes.

Regardless of the different possible causal pathways, sufficient evidence has accumulated to recommend specifically targeting mental health difficulties and low mental wellbeing in caregivers of children with autism. This may require service providers to include a direct focus on caregivers, such as providing advice on parenting skills, offering emotional and practical support, and opportunities for respite or psychological counselling. In the Bromley et al. study (2004), for example, almost all mothers reported a need for more help in the following areas: breaks from child-care (particularly during the holidays); doing things the parent enjoys; and advice on the best ways to help their child. There is also a need to address the child’s functional abilities in the home, and their emotional and behaviour problems, since effective intervention strategies in these areas are available in children with autism (Scahill et al. 2016; Postorino et al. 2017). There is also evidence that such programmes have a positive impact on parental mental health (Tonge et al. 2006). Addressing these essential unmet needs within comprehensive intervention models could substantially improve parental psychological outcomes which, in turn, may further reduce child behaviour problems (Totsika et al. 2013).
